# Modified Use of a Fibular Strut in the Reduction and Stabilization of 2-Part Osteoporotic Proximal Humerus Fractures

**DOI:** 10.5435/JAAOSGlobal-D-20-00153

**Published:** 2020-10-16

**Authors:** Frank R. Avilucea, Kareem Shaath, Ryan Kozlowski, Nima Rezaie

**Affiliations:** From Orlando Health Orthopaedic Institute, Orlando, FL.

## Abstract

**Purpose:**

This study introduces a modified use of a fibular strut allograft as an adjunct to lateral locked plating in the treatment of osteoporotic two-part fractures of the proximal humerus.

**Methods:**

A prospective series of 13 consecutive patients (mean age 68; range, 60 to 88) with displaced two-part fractures of the proximal humerus were included. The main outcome measures included radiographic healing, clinical and radiographic findings of complications, assessment of shoulder function measured with the Shoulder Function Index, and ultrasonography assessment of rotator cuff disruption.

**Results:**

At postoperative month four, every fracture healed as evidenced on radiographic assessment. Clinically, patients achieved an average shoulder forward flexion of 141.5°, external rotation of 37°, and abduction of 98°. The mean Shoulder Function Index score was 73.2 (range, 64 to 77). No patients were included who required a major or a minor revision surgery. The average follow-up was 13.2 months (range, 12 to 15). Ultrasonography demonstrated no tears of the rotator cuff.

**Discussion:**

In a series of 13 patients, our technique facilitated fracture reduction while avoiding additional soft-tissue dissection at the fracture site and enabled supplementary stabilization after application of a lateral locking plate. Using this technique, we had minimal complications, a high rate of osseous healing, and achieved favorable clinical outcomes in a challenging patient population.

Fractures of the proximal humerus account for approximately 6% of all adult fractures and are the third most common injury in patients older than the age of 60 years.^1^ Recent data suggest a notable increase in the numbers of these fractures, primarily because of osteoporosis and the aging population.^2^ Although many of these injuries may be successfully treated nonoperatively, instances exist when surgical stabilization is necessary, a procedure that has high complication rates with locking plate and screw fixation, particularly in the setting of poor bone quality.

First introduced for the treatment of proximal humeral nonunions,^3^ the use of a fibular strut allograft has been used in the primary treatment of fractures of the proximal humerus.^4^ This technique involves placement of a fibular strut allograft within the medullary space to support the medial column and minimize the potential for varus collapse.^4^ The technique has also been reported in the treatment of complex fractures extending into the metadiaphysis to augment plate and screw fixation, permitting an aggressive postoperative rehabilitation program.^5^

Predicated on the effectiveness of an intramedullary fibular strut to augment overall fracture stability, we modified the technique to gain control of the subchondral bone of the humeral head and the lateral corticocancellous bone of the greater tuberosity. This will assist with fracture reduction of two-part osteoporotic fractures and help prevent varus collapse by augmenting plate/screw fixation permitting early active range of motion. Our primary hypothesis was that our technique would help prevent early collapse around a stable implant, prevent implant loosening and loss of fixation, and reliably result in radiographic union without displacement.

## Methods

A prospective database of all proximal humerus fractures that presented to our Level I trauma center during a 20-month period (May 2018 to December 2019) was completed for patients who underwent open reduction and internal fixation with our modified fibular strut technique. Institutional review board approval was obtained for this study. The primary indication for this technique is a two-part osteoporotic fracture of the surgical neck with a minimum of 67% displacement.^6^ In addition, each patient who underwent surgical fixation was independent with all activities of daily living, had no reported dementia, was not an alcoholic, and would be able to participate with postoperative physical therapy. Finally, as our technique involves passage of the fibular strut through the articular surface of the humeral head, head-split or tuberosity fractures were excluded to enable the fibular strut to support the humeral head. A preoperative CT was obtained on every patient to ensure that these criteria were met. Patients with previous surgery to the affected shoulder were excluded as were patients with a previous history of shoulder pain and/or difficulty with arm elevation or abduction. Demographic characteristics including age, sex, smoking status, and medical comorbidities were recorded.

Fracture healing was evaluated both radiographically and clinically by assessing for pain with axial loading of the shoulder. Clinical evaluation at every postsurgical office visit included the presence of superficial or deep infection, shoulder pain using the visual analog scale, and shoulder range of motion (forward elevation, external rotation, and abduction) measured with a goniometer. A bone density scan (dual energy X-ray absorptiometry) was obtained within six weeks of surgery. To assess each patient's ability to do activities involving the affected shoulder, the Shoulder Function Index (SFInX) was obtained.^7^ At the 1-year postoperative mark, each patient's rotator cuff was assessed with ultrasonography.

### Surgical Technique

Patients may be positioned supine or in the beach chair position. We prefer the supine position on a radiolucent table with C-arm positioned on the contralateral side. A standard anterolateral approach using a deltoid-split is completed to enable visualization of the rotator cuff musculature. To gain control of the humeral head, a braided suture is passed through the rotator cuff tendons to facilitate manipulation of the humeral head. Using a blade, the rotator cuff tendon is split in-line with its fibers to permit visualization of the cranial portion of the articular surface. Determining the location of the rotator cuff split may be aided with fluoroscopy by passing a 2.0-mm wire through the rotator cuff onto the humeral head to ensure that the split is located where the fibular strut will enter the humeral head. It is important to avoid injury to the rotator cuff's insertion site or the long head of the biceps tendon, which may be visualized through the split. Finally, no soft-tissue dissection is done at the level of the fracture site.

**Figure 1:**
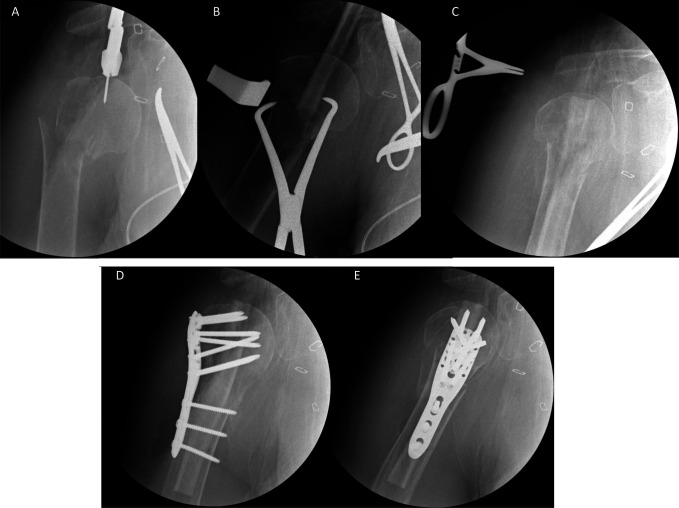
Radiograph demonstrating the starting point that is localized at the cranial aspect of the humeral head with a 2.0-mm Kirschner wire (**A**). The wire is advanced approximately 1-cm. A reamer is positioned over the wire and manipulated by hand to create the entry portal for the fibular strut (**A**). The strut is advanced through the humeral head; on advancing it to the fracture site, a reduction is achieved (**B**) to facilitate passage into the distal segment (**C**). Standard lateral locking plate fixation is thereafter applied (**D** and **E**).

**Figure 2:**
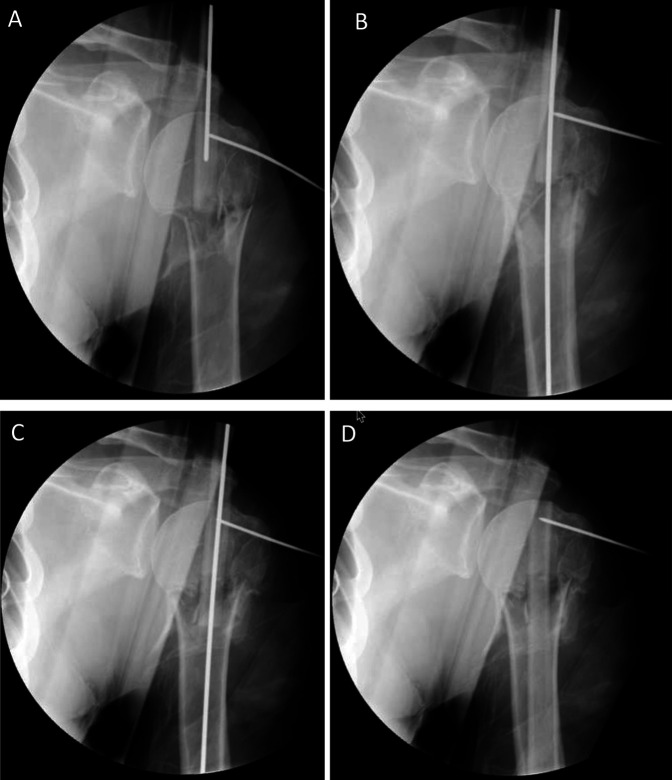
Radiograph demonstrating the cases where fracture site reduction is difficult, the blunt-end of a 2.5-mm ball-tipped wire may be passed through the medullary canal of the fibula (**A**) and into the medullary canal of the distal segment (**B**). The fibular strut may then be gently advanced into the distal segment (**C** and **D**).

A freeze-dried fibular strut measuring 9 to 12 cm in length (sufficiently long to engage the proximal diaphysis) is selected. On the back table, the diameter of the fibular strut is measured and a reamer that is 1-mm greater in diameter is used. A 2.0-mm wire is placed at the most cranial position of the humeral head (Figure 1, A); fluoroscopy is used to ensure appropriate positioning of the wire on both AP and lateral views. Because of potentially osteoporotic bone quality, the reamer is inserted by hand to create an opening in the humeral head (Figure 1, A). Care is taken to ensure that the rotator cuff tendon is not injured by the reamer. The fibular strut is then passed into the humeral head at which point the proximal segment may be manipulated with either the rotator cuff sutures or a pointed-clamp to enable passage of the strut into the humeral shaft (Figure 1, B). In some instances, the blunt-end of a standard 2.5-mm ball-tipped guidewire may be inserted through the fibular strut and into the distal humeral segment to facilitate passage of the cortical strut into the medullary canal of the humeral shaft (Figure 2). With the use of a bone tamp, the strut is advanced until it is approximately 2- to 3-mm caudal to the articular surface of humeral head and contained within the subchondral bone of the humeral head and the corticocancellous bone of the greater tuberosity, a site that helps maintain the reduction achieved with the fibular strut.

The insertion of a lateral locking plate uses the deltoid split to gain access to the humeral head; the axillary nerve is gently palpated to ensure the plate is positioned deep to the nerve. A three- or five-hole locking proximal humerus plate (Synthes) is applied, centered, and provisionally held in place with Kirschner wires. The wires are used to insure the calcar fixation will be appropriate and then distal cortical screws percutaneously inserted into the shaft aiming to obtain quadricortical fixation (bicortical through the humeral shaft and the fibular strut), followed by insertion of locking screws into the head. In every case, calcar screws were placed to optimize fixation. Braided sutures (#2 Ethibond; Ethicon) were passed through the rotator cuff tendons to help manipulate the proximal segment; each of these sutures were passed and fastened to the plate at the conclusion of the case. After inserting screws and irrigating the fibular insertion site with saline, the split in the rotator cuff is repaired with a braided suture.

#### Postoperative Protocol

Each patient was immediately permitted full active and passive shoulder range of motion postoperatively. A sling was provided with the recommendation that it be used for comfort only and discontinued at the end of postoperative week 2. Weight-bearing was protected for four weeks. At the second postoperative check (typically postoperative week 6), each patient commenced progressive strengthening of the arm and shoulder for an additional 6 weeks.

### Statistical Analysis

Data were collected in a Microsoft Excel 2016 database. Descriptive statistics were calculated for demographic characteristics, range of motion, complications, and patient outcomes (SFInX).

## Results

The surgical technique was used to treat 15 patients with completely displaced two-part proximal humerus fractures. Of the 15 patients, two did not follow-up because they were from out of state. Of the remaining 13 patients, the average age was 68 years (range, 60 to 80 years). Eleven patients were women; demographics are noted in Table 1. Nine patients sustained the injury due to a ground-level fall, and the remaining five were involved in a motor vehicle crash. Each patient's fracture was stabilized with a fibular strut and proximal humerus locking plate (Synthes).

**Table 1:** Patient Demographics

Comorbidity	No. of Patients
Hypertension	8
Controlled diabetes mellitus	6
Uncontrolled diabetes mellitus	2
Cardiovascular disease	6
Smoker	6

From an surgical standpoint, seven cases required the use of ball-tipped guidewire to help facilitate passage of the fibular strut into the diaphysis of the humeral shaft. In two cases, pointed clamps were used to help manipulate the humeral head so that an appropriate starting point on the humeral head could be obtained in addition to facilitating passage of the strut into the distal segment. In every case, the strut resisted a varus malreduction once it was inserted. Of note, a size 12-mm reamer was used in 12 cases to create the opening for the strut to enter the humeral head; in one case, a 13-mm reamer was used.

Of the 13 patients included in our analysis, the average final length of follow-up was 13.2 months (range 12 to 15 months). The average bone density T-score was—2.6 (range, 2.5 to 2.8). By postoperative month 4, each patient achieved radiographic evidence of osseous union. In addition, patients had no pain with axial loading through the proximal humerus. At the final follow-up, no radiographic signs of implant loosening, intra-articular screw penetration, or osteonecrosis of the humeral head were found. The average SFInX score was 73.2 (range, 64 to 77) (Table 2). The average final visual analog scale score was 1.1 (range, 0 to 3). Ultrasonography assessment of each patient's rotator cuff demonstrated no tears.

**Table 2:** Patient Characteristics and Clinical Outcomes

Patient No.	Age (yr)	Sex	Mechanism	Fracture Displacement (%)	Follow-Up (mo)	SFInX Score	Forward Flexion	External Rotation	Abduction	Complication	DEXA T-Score
1	72	Female	Fall	80	14	73	140	30	95	None	**−2.6**
2	64	Female	Fall	>100	13	73	130	35	100	None	**−2.8**
3	67	Female	MVC	75	13	77	140	45	105	None	**−2.5**
4	75	Male	Fall	90	13	64	140	40	85	None	**−2.5**
5	78	Female	Fall	100	12	77	150	40	100	Superficial infection	**−2.5**
6	63	Female	Fall	100	15	67	130	30	105	None	**−2.7**
7	70	Female	MVC	>100	12	77	140	40	95	None	**−2.7**
8	72	Female	MVC	>100	12	70	150	40	85	None	**−2.5**
9	80	Female	Fall	75	13	73	140	30	95	None	**−2.5**
10	61	Male	Fall	90	13	77	150	30	105	None	**−2.8**
11	62	Female	MVC	>100	12	70	140	45	95	Superficial infection	**−2.7**
12	60	Female	Fall	80	14	77	140	30	100	None	**−2.6**
13	72	Female	Fall	>100	15	77	150	50	105	None	**−2.8**

DEXA = dual energy X-ray absorptiometry, MVC = motor vehicle crash, SFInX = Shoulder Function Index

**Figure 3:**
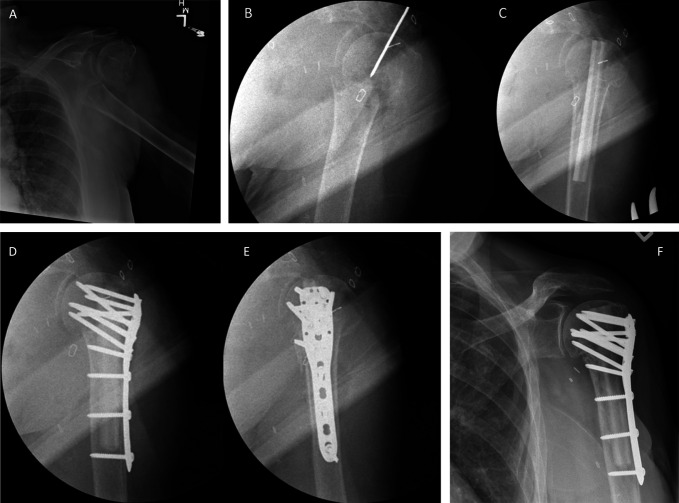
Radiograph demonstrating the case example of a 72-year-old woman who sustained a fall with a resultant displaced proximal humerus fracture of the left upper extremity (**A**). At presentation, the patient also had a pulseless hand. After an emergent revascularization of the axillary artery, stabilization of the fracture was completed using our technique (**B** through **E**). At one year, the patient demonstrated no findings of osseous collapse and maintained stable internal fixation (**F**).

Two patients sustained a traumatic axillary artery injury requiring revascularization. In each patient, the revascularization procedure was completed before fixation of the proximal humerus. The vascular procedure was completed through a separate axillary-based incision. No complications were noted in either patient. Figure 3 provides an example of one of these patients. Two patients were present in our series who developed a superficial surgical site infection; both infections resolved after a short course of oral antibiotics. Neither of these two aforementioned patients smoked tobacco or were diabetic.

## Discussion

Proximal humerus fractures are the third most common fracture in the elderly.^1^ Although the incidence of proximal humerus fractures has not changed, the rate of surgical treatment has increased notably.^8^ The goal after surgical fixation is to allow early range of motion, an important postoperative factor that maximizes the potential for a good clinical outcome.^9-11^ Although many of these fractures may be managed nonoperatively, particularly in the elderly, instances where surgical treatment will yield an improved clinical outcome if complications are avoided.^12,13^

The use of a fibular strut as an adjunct in the treatment of proximal humerus fractures has been well described in a variety of settings. Initially, Gardner et al^4^ reported its effectiveness in supporting medial column corticocancellous loss in a series of seven patients. Subsequent clinical applications further report on its effectiveness as an adjunct in the treatment of complex fractures involving diaphyseal extension.^5^ From a biomechanical standpoint, Mathison et al^14^ demonstrated that using a fibular allograft strut increased loads to failure and construct stiffness while reducing motion at the fracture site. Finally, Neviaser et al^15^ reported on a series of 38 patients in whom a fibular strut was used in combination with a lateral locked plate for the treatment of three- and four-part fractures. The authors noted zero cases of screw cutout or articular screw perforation; however, one case of partial head osteonecrosis was noted which was managed conservatively. Although our series focused on the osteopenic two-part fracture, our results corroborate those of the aforementioned studies such that we had no implant-related complications and achieved fracture healing in every patient.

Our technique uses the fibular strut to capture subchondral bone in the humeral head and corticancellous bone of the greater tuberosity while avoiding a large dissection around the fracture site. The medial start point for the strut facilitates an anatomic alignment; as the strut contacts the diaphysis, an indirect reduction of the proximal fragment occurs in addition to resisting varus collapse. The spanning of the cranial 9 to 12 cm of the proximal humerus with the strut permits application of a laterally locked plate with quadricortical screw insertion in the shaft and several fixed angle points of fixation, incorporating the medullary cortical strut proximally. Finally, by including locked fixation into the strut, a shorter lever arm for the locking screws is imparted. A notable difference in our technique, however, is that that we do not focus on recreating the medial buttress with the strutm as has been discussed in previous reports.^4,16^ Although every patient in our series had some degree of medial column fragmentation, the technique enabled appropriate alignment in both the coronal and sagittal plane, medial cortex apposition, and appropriate calcar screw position. Furthermore, our goal was to use the technique to avoid varus malreduction, a factor that is associated with early loss of fixation, regardless of patient age or fracture pattern.^17^

The SFInX offers a reliable tool to interpret how a patient's function is impacted by their injury and treatment by measuring their activity limitations.^7^ Developed specifically for proximal humerus fractures,^7,18^ the SFInX provides an objective test to determine whether pain is preventing function for the patient, rather than the patient's interpretation of how pain is affecting functionality, and is the recommended clinician measured functional outcome tool in a recent systematic review.^19^ Our patients have shown excellent scores with the SFInX, indicating a return to activities of daily living with minimal limitation after surgical fixation with our technique.

The decision to treat proximal humerus fractures surgically remains controversial because of notable variations in fracture patterns, techniques, and patient factors. The two most common concerns after surgical management are varus malunion and proximal screw cutout. A study done by Bjorkenheim with 72 patients treated with a laterally locked proximal humerus plate showed a 26% rate of varus malunion.^20^ A similar study by Owsley and Gorczyca^21^ in elderly patients showed a varus malunion rate of 25% and a hardware cutout rate of 23%. These complications lead to poor functional outcomes and potential need for revision surgery, with a revision surgery rate of approximately 14%.^22^ Our technique aims to prevent these modes of failure with the fibular strut helping establish anatomic alignment and supplementing overall poor bone quality to prevent screw cutout.

Several limitations exist to our technique and results. To begin, no direct comparison groups of patients exist who were managed with either nonsurgical management or surgical fixation without a fibular strut. As such, we are unable to provide radiographic and functional outcome data to assess how our reported technique would compare with commonly used treatments. As we demonstrate, however, the technique yielded overall acceptable radiographic and functional results with minimal complications. Second, the determination of physical examination findings and shoulder function outcome scores were determined by the treating physician. Although this is the most effective way of determining consistent findings regarding clinical analysis, a potential exists for observer bias. Third, the insertion of the fibular allograft in our series involves splitting the rotator cuff to visualize the start point. Although this entry portal is used, care is taken to not disrupt the rotator cuff insertion site or tendon during the procedure, and a side-to-side repair is completed at the conclusion of the case. In our series, no patients with limited function due to rotator cuff dysfunction were found, and ultrasonography assessment demonstrated an intact tendon. Fourth, although we have shown an effective use of the strut as an adjunct, its presence presents an added challenge should a patient require conversion to arthroplasty, a factor that should be considered before undertaking our described technique. Finally, follow-up in our series is limited to a minimum of 12 months precluding our detection of osteonecrosis of the humeral head should it occur beyond the time frame of our study. Previous studies reporting the on the radiographic outcome of two-part fractures note no radiographic findings of osteonecrosis at the final follow-up (range, 24 to 72 months) if not seen at 1 year.^23,24^

Our clinical series, although retrospective, offers data and a modified technique to the current discussion regarding management of proximal humerus fractures. We report favorable radiographic and clinical outcomes in an elderly population after modified use of the fibular strut to incorporate subchondral bone of the humeral head as a point of stability, avoid further soft-tissue disruption around the fracture site, facilitate fracture reduction, and augment plate fixation.
